# Antimicrobial Prescription Pattern in Ho Teaching Hospital, Ghana: Seasonal Determination Using a Point Prevalence Survey

**DOI:** 10.3390/antibiotics10020199

**Published:** 2021-02-18

**Authors:** Cornelius C. Dodoo, Emmanuel Orman, Thelma Alalbila, Adelaide Mensah, Jonathan Jato, Kwadwo A. Mfoafo, Isaac Folitse, Araba Hutton-Nyameaye, Inemesit Okon Ben, Paapa Mensah-Kane, Emmanuel Sarkodie, Eneyi Kpokiri, Misha Ladva, Benedict Awadzi, Yogini Jani

**Affiliations:** 1 School of Pharmacy, University of Health and Allied Sciences, PMB 31, Ho, Ghana; eorman@uhas.edu.gh (E.O.); talalbila@uhas.edu.gh (T.A.); amensah@uhas.edu.gh (A.M.); jjato@uhas.edu.gh (J.J.); kmfoafo@uhas.edu.gh (K.A.M.); aharaba@uhas.edu.gh (A.H.-N.); ioben@uhas.edu.gh (I.O.B.); pmensah-kane@uhas.edu.gh (P.M.-K.); 2 Ho Teaching Hospital, P.O. Box MA 374, Ho, Ghana; ifolitse@yahoo.com (I.F.); benedictawadzi@gmail.com (B.A.); 3 Department of Pharmacy, Kwame Nkrumah University of Science and Technology Hospital, University Post Office, KNUST, Kumasi, Ghana; emmsarkodie@knust.edu.gh; 4 Department of Clinical Research, Faculty of Infectious and Tropical Diseases, London School of Hygiene and Tropical Medicine, London WC1N 1AX, UK; Eneyi.Kpokiri@lshtm.ac.uk; 5 Department of Practice and Policy, University College London School of Pharmacy, London WC1N 1AX, UK; 6 Joint Research Office, University College London, London WC1E 6BT, UK; misha.ladva@ucl.ac.uk; 7 Centre for Medicines Optimisation Research and Education, University College London Hospitals, NHS Foundation Trust, London NW1 2BU, UK

**Keywords:** point prevalence survey, developing countries, antimicrobial stewardship

## Abstract

A standardised Global Point Prevalence Survey (PPS) tool was used to determine the antimicrobial prescription pattern in the Ho Teaching Hospital on two separate occasions in a total of 14 wards in the hospital, including dedicated wards for paediatrics and neonates. Manually collected and anonymised data were entered, validated, analysed and reported using a web-based global PPS application. With 147 and 153 patients considered in the July 2019 and January 2020 surveys, respectively, 98 patients (66.7%) and 84 patients (54.9%) had received one or more antimicrobials. The prevalence of antimicrobial use in the adult wards was 64.3% (72/112) and 53.4% (63/118) in the first and second surveys, respectively. The prevalence in the paediatric wards was 60.0% (12/20) and 62.5% (10/16), respectively, in the two surveys, while that in the neonatal wards was 93.3% (14/15) and 57.9% (11/19), respectively. β-lactams were the most used antibiotics in both periods. Malaria was the most common diagnosis requiring the use of antimicrobials in July 2019, accounting for 19.4% of the diagnoses, whereas in January 2020, it was skin and soft-tissue conditions (28.1%). This reflects a seasonal association between malaria and rainfall patterns. Out of the antimicrobials prescribed during each of the survey periods, 95% were used for empirical treatment, and this could be attributed to a number of reasons, including logistical challenges, among others, that require further exploration in the context of local, national and international policy recommendations.

## 1. Introduction

Inappropriate antibiotic prescribing is a major public health threat globally. Antibiotic consumption in low- and middle-income countries (LMICs) is on the rise because of rapid economic and population growth coupled with the high burden of infectious diseases [1,2]. There is little knowledge about the current resistance patterns of common pathogenic bacteria in sub-Saharan Africa, where surveillance capacity is minimal [3]. Surveillance systems to monitor antimicrobial use and resistance are needed to improve decision making and assess the effect of interventions [4,5]. Continuous surveillance, especially prospective active surveillance, is thought to be the gold standard [6]. Since 2015, the Global Point Prevalence Survey offers a simple approach to assess antimicrobial consumption and resistance patterns in hospitalised patients across regions and countries worldwide [7]. This Global Point Prevalence Survey (Global-PPS) of antimicrobial consumption and resistance aims to assess the global prevalence of antimicrobial use and resistance, with emphasis on countries with low resources, support and expertise, like Ghana. Global-PPS is designed to systematically collect granular and representative data to inform government and international agencies on policies around optimising antibiotic prescribing and minimising antibiotic resistance [8].

This study sought to determine the antimicrobial prescription pattern in the Ho Teaching Hospital (HTH) using a standardised Global Point Prevalence Survey (PPS) tool.

In this work, we report two surveys that were carried out in July 2019 and January 2020, which are representative of the two different seasons observed in the country, to identify any trends or differences in prescription patterns over these periods. Between the first and second PPSs, training on antimicrobial stewardship was provided, and the results from the first survey were presented to staff of the teaching hospital to identify areas of improvement. In furtherance of this, management of the hospital has established a working antimicrobial stewardship team.

## 2. Results

### 2.1. Prevalence of Antibiotic Prescriptions

**Figure 1:**
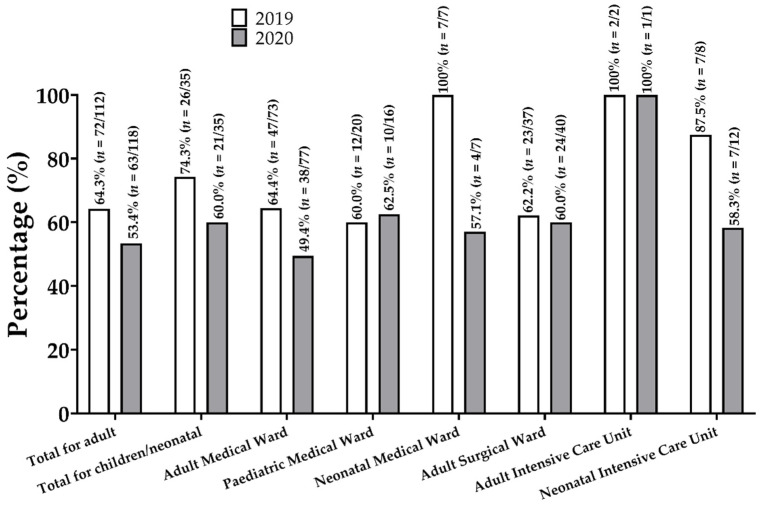
Antimicrobial prevalence in the Ho Teaching Hospital in July 2019 and January 2020. The numbers in the parentheses are expressed as follows: number of patients receiving one or more antimicrobials/number of patients admitted in the respective ward.

The prevalence of antimicrobial prescriptions for hospital inpatients was 66.7% (*n* = 98/147) and 54.9% (*n* = 84/153) in July 2019 and January 2020, respectively. In July 2019, the antimicrobial prevalence was 64.3% (*n* = 72/112) among adults and 74.3% (*n* = 26/35) among children and neonates. On the other hand, the antimicrobial prevalence in January 2020 was 53.4% (*n* = 63/118) among adults and 60.0% (*n* = 21/35) among children and neonates. Within the various wards considered, there were differences in the prevalence of the use of antimicrobials in the two surveys. For example, while the prevalence was 64.4% (*n* = 47/73) in the adult medical ward in the first survey, the prevalence reduced to 49.4% (*n* = 38/77) in the second survey. In addition, while the prevalence in the neonatal intensive care unit was 87.5% (*n* = 7/8) in 2019, the prevalence declined to 58.3% (*n* = 7/12) in 2020. In summary, the highest prevalence of antimicrobial use across the two periods was observed in the neonatal medical ward (100%, *n* = 7/7) in the 2019 survey and the adult intensive care unit in both 2019 and 2020 (100%, *n* = 2/2; 100, *n* = 1/1). The details on the prevalence in the surveyed wards are illustrated in Figure 1.

### 2.2. Types of Antimicrobials Prescribed

**Figure 2:**
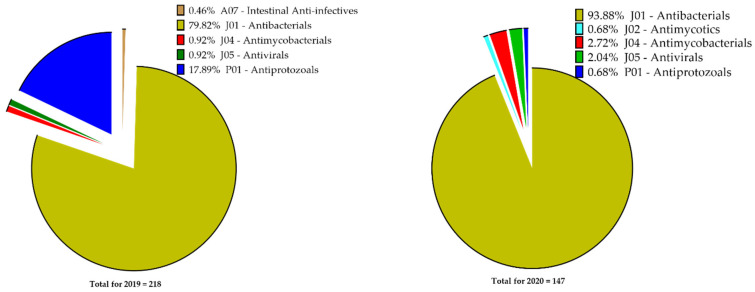
Classes of antimicrobials used in the Ho Teaching Hospital in July 2019 and January 2020.

In the July 2019 survey, there were 218 antimicrobial prescriptions across six different Anatomical Therapeutic Chemical (ATC) classifications, while the January 2020 survey recorded 147. Four of the classes, Antibacterials for Systemic Use (J01), Antimycobacterials (J04), Antivirals (J05) and Antiprotozoals (P01), were prevalent in both surveys. The fifth class in the 2019 survey was the Intestinal Anti-infectives (A07) and in the 2020 survey Antimycotics (J02) (Figure 2). In both surveys, the Antibacterials for Systemic Use class formed the majority, with 79.8% (*n* = 174/218) in 2019 and 93.9% (138/147) in 2020. The proportions of antimicrobials within the classes were significantly different in each year (*χ*^2^ = 30.46, *p* < 0.0001), as shown in Table 1.

In considering the antibacterials for systemic use, penicillins and other β-lactams (mostly cephalosporins) were the most prescribed (Table 1). The use of extended-spectrum penicillins and β-lactamase-resistant penicillins increased from 2019 to 2020, whereas the opposite was observed for β-lactamase-sensitive penicillins. Most of the cephalosporins prescribed were of the second and third generations, with no records of the use of first-generation cephalosporins in both periods and minimal use of carbapenems in 2019 (1.5%, *n* = 1/68) (Table 1). The use of other broad-spectrum antibiotics such as aminoglycosides and tetracyclines was low during the two surveys. Other antibacterials (17.8%, *n* = 31/174) were also recorded, and these were mostly parenteral metronidazole prescribed for post-surgical prophylaxis in gynaecological-, gastrointestinal- and orthopaedic-related cases, sepsis and pneumonia.

### 2.3. Common Diagnoses for Antimicrobials Use

**Figure 3:**
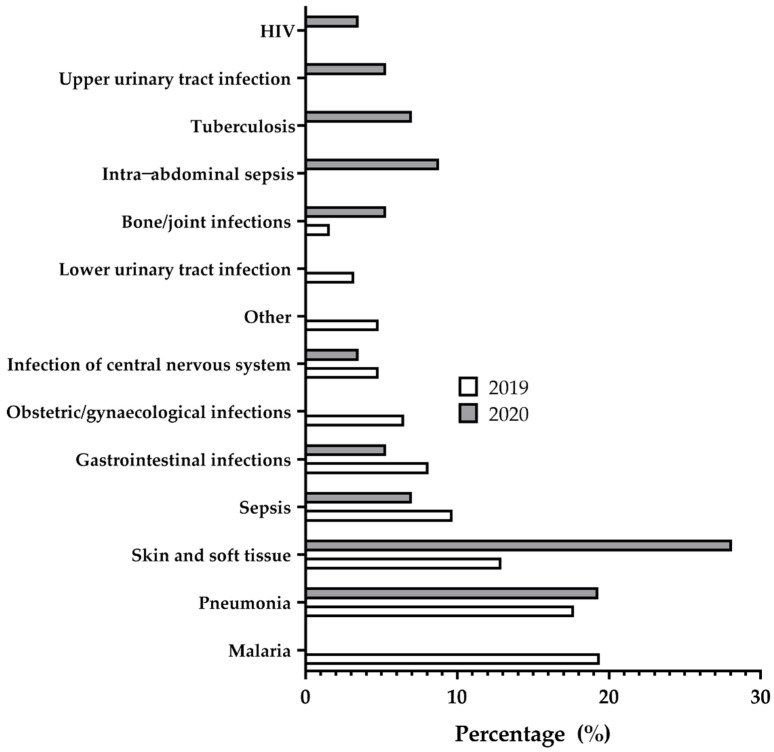
Most common diagnoses treated with therapeutic antimicrobials in July 2019 and January 2020. These figures are in reference to patients; hence, a patient with multiple diagnoses can be counted several times.

In 2019, malaria (19.4%, *n* = 12) was the most common diagnosis requiring the prescription of antimicrobial drugs in the hospital. This was followed by pneumonia (17.7%, *n* = 11) and skin and soft-tissue infections (12.9%, *n* = 8). In 2020, skin and soft-tissue infections (28.1%, *n* = 16) were the most common diagnosis for antimicrobials, followed by pneumonia (19.3%, *n* = 11) and intra-abdominal sepsis (8.8%, *n* = 5). There were notable differences in diagnosis between the two survey periods. Malaria was not a common diagnosis in the 2020 survey. Lower urinary tract infections (3.2%, *n* = 2) and obstetric and gynaecological infections were common in the July 2019 survey; and intra-abdominal sepsis (8.8%, *n* = 5), tuberculosis (7.0%, *n* = 4), upper urinary tract infections (5.3%, *n* = 3) and human immunodeficiency virus disease (3.5%, *n* = 2) were also common in the January 2020 survey (Figure 3).

### 2.4. Patterns of Antibiotics Use

In both surveys, it was observed that majority of the antibiotic prescriptions in the HTH were empiric, with an average of 95.8% and 88.9%, respectively, for July 2019 and January 2020 surveys.

Community-acquired infections (CAI) accounted for 90.7% and 77.9% of all antibiotics prescribed in the 2019 and 2020 surveys, respectively, while healthcare-associated infections (HAI) accounted for 9.3% and 22.1%, respectively. Prophylactic use of antibiotics was mainly for pre-surgical purposes, constituting 79.4% and 62.7% for the two surveys, respectively, whereas medical prophylaxis was 20.6% and 37.3%, respectively.

In the management of sepsis in both adults and children, it was observed that eight antibiotics belonging to five groups were prescribed to six patients in the 2019 survey, while this was narrowed to four antibiotics belonging to three groups prescribed to four patients in the 2020 survey (Table 2). In the 2019 survey, two out of six patients received a combination of two antibiotics each (that is, metronidazole/ceftriaxone and cefuroxime/ceftriaxone), while each of the remaining four received a single antibiotic. Similarly, two of the four patients in the 2020 survey also received combinations of metronidazole and ceftriaxone, while the other two independently received ceftriaxone and gentamicin. There was minimal change in the management of pneumonia, with amoxicillin, azithromycin, ceftriaxone and a combination of sulfamethoxazole and trimethoprim commonly used in both periods. The other antibiotics used included cefuroxime in 2019 and metronidazole in 2020 (Table 2). In the 2019 survey, six antibiotics were equally used for medical prophylaxis, but the number reduced to three in the 2020 survey. These medicines were mainly used to prevent pneumonia infections.

### 2.5. Quality Indicators for Prescribing

Whereas most of the treatments were empiric, as indicated above, the study revealed that most prescribers stated the reason for the prescription of antimicrobial agents and most of the prescriptions were guideline-compliant during both periods. There was an increase in the reasons in medical notes from an average of 82.3% to 97.9% and guideline compliance from 73.8% to 81.8% between the 2019 and 2020 surveys, respectively.

There was a stop or review date stated in almost all the antimicrobial drug prescriptions in both surveys, revealing strict compliance to this quality indicator (Table 3).

## 3. Discussion

### 3.1. Prevalence

These point prevalence surveys were the first-ever large-scale surveys on antimicrobial use conducted in the HTH. It gave an insight into the antibiotic prescription patterns in the facility, and since the surveys were conducted at different seasons in Ghana, we were able to ascertain whether the prescription patterns vary during these different periods. Overall, the prevalence of antimicrobial prescriptions for inpatients was 66.7% and 54.9% in July 2019 and January 2020, respectively, with more antibiotics used for children and neonates compared to adults in both seasons. Higher rates of antimicrobial use are usually associated with the inappropriate use and development of antimicrobial resistance and healthcare-associated infections [9,10]. It is therefore imperative for antimicrobial use to be justified. Of the patients surveyed, 79.7% as compared to 46.5% were on parenteral antimicrobials and 64.9% as compared to 39% on multiple-antimicrobial therapy for the 2019 and 2020 surveys, respectively.

The prevalence from this study was comparable to similar data from two other teaching hospitals in Ghana, the Komfo–Anokye Teaching Hospital and the Korle-Bu Teaching Hospital with a prevalence of 64.0% and 51.4%, respectively [11,12]. A study conducted in the Keta Municipal Hospital, also in Ghana, showed a significantly higher prevalence of 82.0% [13]. A PPS report on a tertiary hospital in Nigeria indicated an antimicrobial prevalence of 78.2% [14], and another report in East Africa reported 67.7% [15].

### 3.2. Types of Antibiotics Prescribed

It was observed that β-lactams were the most prescribed antibiotic class in the HTH. The dominance of β-lactam use in comparison to other classes of antimicrobials is not a new trend. Similar patterns were observed in Komfo Anokye Teaching Hospital, the Keta Municipal Hospital, and the Ghana Police Hospital [11,13]. The high use of cephalosporins in the HTH could be attributed to the fact that the majority of the treatments in the HTH are empiric. The absence of prescriptions for first-generation cephalosporins within the facility was similar to a report from the survey conducted in the Korle Bu Teaching Hospital as well [12]. The first-generation cephalosporins are currently not covered by the National Health Insurance Scheme (NHIS), a health insurance scheme introduced to provide financial access to healthcare for all Ghanaian residents, a possible reason why they were not prescribed. Most of the clients who attend this facility have subscribed to the NHIS and were probably unwilling to pay for medicines that were not covered by this scheme [16]. In the era of antimicrobial resistance, it is important to limit the use of broad-spectrum antimicrobials and rather opt for limited-spectrum antimicrobials that are still efficacious. A recent study in Japan demonstrated the presence of some first-generation cephalosporins in use in some hospitals [17] and proposed that this class of medicines be reconsidered if available evidence proves their efficacy.

The recurrence of ceftriaxone and metronidazole in both periods may indicate the preference of their use in the management of sepsis in the hospital. The use of cefuroxime, ciprofloxacin and meropenem in 2019 was replaced with gentamicin in 2020. A meeting with the physicians after the second survey indicated that there was a decision to restrict the use of cefuroxime, ciprofloxacin and meropenem. This was apparent in the 2020 survey results, with the reduction in the usage of these antibiotics. The low use of carbapenems, which belong to the watch or reserve categories of the 2019 World Health Organisation AWaRe classification of antibiotics [18], indicates the hospital’s commitment to safeguarding the integrity of these medicines.

The basis for the prescription of antibiotics was generally empirical rather than targeted in both surveys. This could be attributed to the limited laboratory resources to aid the diagnosis of specific pathogens, resulting in broad-spectrum empiric treatments aiming to cover all possible pathogens that could be responsible for the infections.

It is worth noting that most of the commonly used antimicrobials are for the right indications; however, the scope of choice was more restricted for surgical prophylaxis than in the case of medical prophylaxis; this was also maintained across the two periods. It is understood that the surgical units of the hospital operate with a local guideline, and this could be the reason for the consistent trend in antimicrobials used.

### 3.3. Commonly Diagnosed Diseases

The most commonly diagnosed disease during the July 2019 survey was malaria, whilst that for January 2020 was skin and soft-tissue infections. The former reflects a seasonal impact, considering that this period was during the rainy season in Ghana, which is characterised by puddles and overgrowth of bushes. The use of antimicrobial agents for skin and soft-tissue infections was similar to the surveys conducted in the Keta Municipal Hospital and the Ghana Police Hospital [13]. Ghana is reported to have around 5000 pneumonia-related deaths annually in children younger than 5 years [19], and as revealed in this study, pneumonia was the second commonest disease condition in both surveys, highlighting the importance of this disease during both seasons.

### 3.4. Quality Indicators

The indicators evaluated in the HTH showed that the facility has several positive practices regarding antimicrobial stewardship. This was evident during both surveys, as reasons for antimicrobial prescriptions were documented in almost 70% of the cases. In addition, there were stop or review dates documented in over 90% of the instances where antimicrobials were prescribed for inpatients. Adherence to guidelines was seen in over 60% of the instances where antimicrobials were prescribed. These were highly commendable and should be encouraged in the promotion of antimicrobial stewardship. Further improvements were noted for documentation of reasons in the medical notes and compliance with guidance, suggesting that awareness and feedback of these indicators were influential.

### 3.5. Implications for Practice

The surveys provided useful insights into the use of antimicrobials at the HTH and an opportunity to benchmark practice against other hospitals in the region and identify areas of focus for improvement. It is hoped that the Antimicrobial Stewardships Committee established as a result of this project will rely on this data and implement interventions, such as retooling of the laboratory and diagnostic services, by increasing manpower, establishing a culture- and sensitivity-testing facility and regularising antimicrobial resistance patterns. This will also inform and support the creation of local guidelines where they are inexistent and continuous conduct of the PPS to evaluate the impact of these interventions. Other stakeholders, including management and the drug and therapeutics of the hospital, have been engaged and educated on the impact of these outcomes and how to improve them.

## 4. Materials and Methods

### 4.1. Study Settings and Design

The PPS was conducted in July 2019 and January 2020 in the HTH. The HTH is located in the capital city of the Volta region and was established in November 1998. The hospital is a tertiary care facility with a staff strength of about 1200, a bed capacity of 306 and 14 wards. It is the main referral facility in the Volta region. The total number of outpatient attendance and inpatient admissions for the year 2019 were 164,173 and 9562, respectively.

All patients on admission at 8:00 a.m. on the day of the survey formed the baseline population of the survey (denominator). However, only folders of inpatients on admission before 8:00 a.m. on the day of the survey who were receiving a systemic antimicrobial agent were included in the study. The departments included in this study were the neonatal intensive care, urology, psychological medicine, gynaecology, isolation, child health, adult intensive care, female surgical, maternity, male surgical, male medical, baby’s unit, female medical, accidents and emergency.

Antimicrobial stewardship training was conducted by pharmacists from the University College London Hospitals NHS Foundation Trust (UCLH, UK) after the July 2019 survey for 60 multidisciplinary health professionals. The aim of the training was to increase their knowledge in antimicrobial stewardship and infection prevention and control (IPC). The participants included physicians, pharmacists, nurses, midwives, biomedical scientists and hospital administrators.

After the conduct of the first Global-PPS, the outcomes were disseminated with the hospital management and the entire health team.

### 4.2. Data Collection

Data were collected on a single day for each survey. A multidisciplinary team of doctors, nurses, biomedical scientists and pharmacists from the University College London Hospitals NHS Foundation Trust (UCLH, UK), the Ho Teaching Hospital (Ghana) and the University of Health and Allied Sciences (Ghana) conducted the survey. Training on the PPS objectives and methodology was conducted for the survey team by pharmacists from the UCLH.

The survey team performed retrospective data collection using patients’ folders and treatment charts to complete standardised case report forms, which comprised a patient-level structured template and a ward data form detailing the specialty of the ward, bed capacity and the number of admitted patients. For patients receiving one or more antimicrobials, data were collected on each antimicrobial prescribed, including the agent, the route of administration, the dosage and indication and diagnosis by the anatomical site in the case of treatment. Basic patient demographics, laboratory data prior to treatment and other quality indicators such as guideline compliance, documentation of reasons for use in medical notes, stop/review date documentation and treatment type were also recorded.

### 4.3. Data Handling and Analysis

Anonymous patient data were collected on paper forms and then entered onto a web-based system for data collection, validation and reporting designed by the University of Antwerp, Belgium (https://www.global-pps.com, accessed on 23 November 2020). Descriptive statistics were used to examine data on antimicrobial use.

## Figures and Tables

**Table 1:** Proportions of antimicrobial classes prescribed over the period of study.

Anatomical Therapeutic Chemical (ATC) Classification	Class/Sub-Class of Antimicrobials	Proportion of Antimicrobials in July 2019	Proportion of Antimicrobials in January 2020
A07—Intestinal Anti-infectives	*Number of Antimicrobials and Patients*	*N* = 1	-
	Antibiotics	100% (*n* = 1/1)	-
J01—Antibacterials for systemic use	*Number of Antimicrobials and Patients*	*N* = 174; 88 patients	*N* = 138; 82 patients
	Tetracyclines	1.1% (*n* = 2/174)	-
	Penicillins	21.3% (*n* = 37/174)	22.5% (*n* = 31/138)
	*a. Penicillins with extended spectrum*	*37.8% (n = 14/37)*	*51.6% (n = 16/31)*
	*b. β-lactamase = sensitive penicillins*	*21.6% (n = 8/37)*	*3.2% (n = 1/31)*
	*c. β-lactamase = resistant penicillins*	*29.7% (n = 11/37)*	*45.2% (n = 14/31)*
	*d. Combinations of penicillins, including β-lactamase inhibitors*	*10.8% (n = 4/37)*	-
	Other β-lactams	39.1% (*n* = 68/174)	26.1% (*n* = 36/138)
	*a. First-generation cephalosporins*	-	-
	*b. Second-generation cephalosporins*	*51.5% (n = 35/68)*	41.7% (*n* = 15/36)
	*c. Third-generation cephalosporins*	*47.1% (n = 32/68)*	58.3% (*n* = 21/36)
	*d. Fourth-generation cephalosporins*	-	-
	*e. Carbapenems*	*1.5% (n = 1/68)*	-
	Sulfonamides/trimethoprim	1.7% (*n* = 3/174)	5.8% (*n* = 8/138)
	Macrolides/liconsamides/streptogramins	8.0% (*n* = 14/174)	13.8% (*n* = 19/138)
	Aminoglycosides	2.9% (*n* = 5/174)	2.2% (*n* = 3/138)
	Quinolones	8.0% (*n* = 14/174)	10.1% (*n* = 14/138)
	Other antibacterials	17.8% (*n* = 31/174)	19.6% (*n* = 27/138)
J02—Antimycotics for systemic use	*Number of Antimicrobials and Patients*	-	*N* = 1; 1 patient
	Triazole derivatives	-	100% (*n* = 1/1)
J04—Antimycobacterials	*Number of Antimicrobials and Patients*	*N* = 2; 2 patients	*N* = 4; 4 patients
	Other drugs for tuberculosis	-	25.0% (*n* = 1/4)
	Combinations of drugs for tuberculosis	100% (*n* = 2/2)	75.0% (*n* = 3/4)
J05—Antivirals	*Number of Antimicrobials and Patients*	*N* = 2; 2 patients	*N* = 3; 1 patient
	Nucleosides and nucleotides, excluding reverse transcriptase inhibitors	50.0% (*n* = 1/2)	-
	Nucleosides and nucleotides reverse transcriptase inhibitors	-	66.7% (*n* = 2/3)
	Combinations of antivirals for treatment of HIV infections	50.0% (*n* = 1/2)	
	Other antivirals	-	33.3% (*n* = 1/3)
P01—Antiprotozoals	*Number of Antimicrobials and Patients*	*N* = 39; 31 patients	*N* = 1; 1 patient
	Nitroimidazole derivatives	53.8% (*n* = 21/39)	-
	Antimalarials	46.2% (*n* = 18/39)	100% (*n* = 1/1)
	*a. Artemisinin and derivatives*—*plain*	*38.9% (n = 7/18)*	100% (*n* = 1/1)
	*b. Combinations of artemisinin and derivatives*	*61.1% (n = 11/18)*	-

**Table 2:** Commonly used antibiotics in selected disease conditions in July 2019 and January 2020.

Disease Condition	Antibiotics Used in July 2019	Antibiotics Used in January 2020
Sepsis in adults and children	***8 antibiotics in 6 patients***	***6 antibiotics in 4 patients***
	Ceftriaxone (50%, *n* = 4/8)	Ceftriaxone (50%, *n* = 3/6)
	Cefuroxime (12.5%, *n* = 1/8)	Metronidazole (33.3%, *n* = 2/6)
	Ciprofloxacin (12.5%, *n* = 1/8)	Gentamicin (16.7%, *n* = 1/6)
	Meropenem (12.5%, *n* = 1/8)	
	Metronidazole (12.5%, *n* = 1/8)	
Pneumonia in adults and children	***15 antibiotics in 11 patients***	***15 antibiotics in 11 patients***
	Amoxicillin (33.3%, *n* = 5/15)	Amoxicillin (54.5%, *n* = 6/11)
	Ceftriaxone (33.3%, *n* = 5/15)	Ceftriaxone (6.7%, *n* = 1/15)
	Azithromycin (20.0%, *n* = 3/15)	Azithromycin (13.3%, *n* = 2/15)
	Cefuroxime (6.7%, *n* = 1/15)	Metronidazole (20.0%, *n* = 3/15)
	Sulfamethoxazole and trimethoprim (6.7%, *n* = 1/15)	Sulfamethoxazole and trimethoprim (13.3%, *n* = 2/15)
Medical prophylaxis in adults and children	***8 antibiotics in 5 patients***	***4 antibiotics in 4 patients***
	Amoxicillin and enzyme inhibitor (12.5%, *n* = 1/8)	Amoxicillin (50.0%, *n* = 2/4)
	Azithromycin (12.5%, *n* = 1/8)	Cefuroxime (25.0%, *n* = 1/4)
	Ceftriaxone (12.5%, *n* = 1/8)	Sulfamethoxazole and trimethoprim (25.0%, *n* = 1/4)
	Cefuroxime (12.5%, *n* = 1/8)	
	Ciprofloxacin (12.5%, *n* = 1/8)	
	Metronidazole intravenous infusion IV (12.5%, *n* = 1/8)	
	Metronidazole oral (12.5%, *n* = 1/8)	
	Sulfamethoxazole and trimethoprim (12.5%, *n* = 1/8)	
Surgical prophylaxis in adults and children	***84 antibiotics in 32 patients***	***30 antibiotics in 19 patients***
	Cefuroxime (35.7%, *n* = 30/84)	Cefuroxime (26.7%, *n* = 8/30)
	Metronidazole (44.0%, *n* = 37/84)	Metronidazole (33.3%, *n* = 10/30)
	Ciprofloxacin (8.3%, *n* = 7/84)	Ciprofloxacin (10%, *n* = 3/30)
	Amoxicillin (6.0%, *n* = 5/84)	Amoxicillin (20.0%, *n* = 6/30)
	Gentamicin (2.4%, *n* = 2/84)	Ceftriaxone (3.3%, *n* = 1/30)
Surgical prophylaxis of the gastrointestinal tract in adults and children	10 antibiotics in 5 patients	8 antibiotics in 4 patients
	Metronidazole (50.0%, *n* = 5/10)	Metronidazole (37.5%, *n* = 3/8)
	Ciprofloxacin (40.0%, *n* = 4/10)	Ciprofloxacin (37.5%, *n* = 3/8)
	Cefuroxime (10.0%, *n* = 1/10)	Amoxicillin (25.0%, *n* = 2/8)

**Table 3:** Summary of quality indicators for antimicrobial prescribing.

	July 2019, *N* (%)			January 2020, *N* (%)			Statistical Parameters
	Medical	Surgical	ICU	Medical	Surgical	ICU	χ^2^, *p*-Value
Reasons in medical notes	62 (68.1)	52 (78.8)	17 (100)	66 (95.7)	50 (98.0)	17 (100)	2.758, 0.2519
Guidelines missing	4 (4.4)	22 (33.3)	0 (0)	6 (8.7)	20 (39.2)	0 (0)	-
Guideline compliant	36 (73.5)	13 (59.1)	8 (88.9)	33 (76.7)	11 (68.8)	8 (100)	0.2510, 0.8821
Stop/review date	86 (94.5)	66 (100)	17 (100)	69 (100)	51 (100)	17 (100)	0.08619, 0.9578

For reasons in medical notes and stop/review dates documented, *N* = number of antimicrobial agents prescribed. ICU: Intensive Care Unit.

## Data Availability

All data generated or analysed during this study have been included in this article.
